# Chlordane-Induced Neurotoxicosis in Urban and Suburban Detroit, Michigan Striped Skunks (*Mephitis mephitis*)

**DOI:** 10.3390/toxics13050367

**Published:** 2025-05-01

**Authors:** Rachel Sheffler, Birgit Puschner, Julie Melotti, Scott D. Fitzgerald, John P. Buchweitz

**Affiliations:** 1 Department of Pathobiology and Diagnostic Investigation, College of Veterinary Medicine, Michigan State University, East Lansing, MI 48824, USA; sheffle5@msu.edu (R.S.);; 2 Veterinary Diagnostic Laboratory, Toxicology Section, Michigan State University, Lansing, MI 48910, USA; 3 Wildlife Disease Laboratory, Michigan Department of Natural Resources, Lansing, MI 48910, USA

**Keywords:** neurotoxicosis, organochlorine, chlordane, persistent organic pollutants, Great Lakes watershed, skunk, wildlife

## Abstract

Despite the ban of technical chlordane, contamination from this persistent organic pollutant has threatened wildlife and human health nearly forty years since its last application. The purpose of this study is to highlight the need for more systemic, broad-scale research efforts to monitor technical chlordane in wildlife sentinel species in urban settings to understand the nature and extent of pesticide pollution and mitigate risk associated with exposure to these compounds. This study presents an unusual finding of neurotoxicosis and elevated chlordane metabolite concentrations in Michigan striped skunks in the absence of other viral or toxic etiologies. In this study, eight of seventeen skunks displaying illness and neurologic signs had brain tissue concentrations of combined oxychlordane, heptachlor epoxide, and trans-nonachlor exceeding the 1000 ng/g wet weight diagnostic threshold for toxicosis. Liver tissue concentrations were ten-fold greater than those of the brain when measured on a lipid weight basis, which can help predict lethal brain residues in skunks. The ongoing presence of chlordane in the environment is expected to cause further unintended consequences for wildlife across the Detroit Metropolitan Area for decades to come. Together, veterinary toxicologists, wildlife biologists, environmental toxicologists, ecologists, and policy makers must utilize a One Health transdisciplinary approach and continue to evaluate the long-term effects of chlordane exposure. As with other pollutants in the River Rouge and River Raisin Areas of Concern, the presence of chlordane in the urban environment presents a significant risk for animal, human, and ecological health.

## 1. Introduction

Across the Great Lakes basin, decades of industrial discharges and agricultural runoff have contaminated soil sediments with persistent organic pollutants including organochlorine pesticides, polychlorinated biphenyls (PCBs), polycyclic aromatic hydrocarbons (PAHs), heavy metals, oil, and grease [1,2]. The vast freshwater ecosystem surrounds the state of Michigan and interconnects with the state’s most urbanized and densely populated watersheds; these watersheds include the River Rouge and River Raisin which span the greater Detroit Metropolitan Area across Wayne, Oakland, and Washtenaw Counties [1,2,3,4]. The River Rouge was designated as an Environmental Protection Agency (EPA) Area of Concern (AOC) under the Great Lakes Water Quality Agreement of 1987. Multiple compounds were banned throughout the 1970s whose manufacture and use contributed to watershed contamination, including PCBs, once used in coolants and lubricants for transformers and electrical equipment, and organochlorine pesticides including chlordane, dieldrin, and dichlorodiphenyltrichloroethane (DDT), previously used in insect control.

Technical chlordane is a synthetic mixture of more than 140 components that was extensively used in the United States until it was banned in 1988 [5]. Due to its chemical stability, chlordane continues to persist in the environment today despite bans on its production and use in the United States and many countries globally following the Stockholm Convention on Persistent Organic Pollutants in May 2001 [5,6]. The composition of technical chlordane is 60% cis- and trans-chlordane and 40% related compounds including heptachlor, trans-nonachlor, cis-nonachlor, chlordene, and other minor constituents [5]. These compounds have environmental half-lives of 10–20 years, are resistant to environmental breakdown, and continue to pose a risk to wildlife and public health [7]. Mammalian, avian, and microbial metabolisms favor the trans-isomer of chlordane and result in the formation of epoxide metabolites, oxychlordane, and heptachlor epoxide, which accumulate in fat [8,9,10,11,12].

Before its ban in the United States in 1988, chlordane was widely used for termite control around the foundation of homes, for the control of scarab beetle populations on golf courses, and for agricultural pest management [5,7,13]. Termiticides applied around foundation walls remained within the upper 6–8 inches of soil without vertical migration to deeper layers [7]. Soil testing has shown low concentrations of oxychlordane and heptachlor epoxide with a greater presence of cis- and trans-chlordane [13,14]. In contrast, significant residues of oxychlordane and heptachlor epoxide have been documented in oriental scarab beetles, other species of beetles, and earthworms [13]. In suburban and urban areas where chlordane was liberally used, it continues to contaminate habitat and food sources for wildlife with pesticide accumulation documented in the tissues of bats [13,15,16], racoons [17,18], cats [19], dogs [19], songbirds [11,13,20,21], raptors [22], and now skunks. Skunks that burrow under porches and areas near foundations while consuming insectivorous diets that include beetles, earthworms, bees, and caterpillars can be predicted to have significant chlordane metabolite residues [23].

Chlordane and its metabolites are reported to cause neurotoxic effects such as tremors, salivation, ataxia, depression, vomiting, and seizures in humans [24,25], mammals [5,26], and birds [11,20]. Specifically, oxychlordane, a metabolite of chlordane, is six times more toxic to birds and heptachlor epoxide, the metabolite of heptachlor, is ten times more toxic to rats than their respective parent compounds [11,13]. Herein, we employed a highly sensitive and selective gas chromatography tandem mass spectrometry (GC-MS/MS) method to detect high concentrations of chlordane metabolites in skunk tissues collected from urban and suburban neighborhoods in Michigan. Our findings provide the first evidence of skunks succumbing to neurotoxicosis by chlordane exposure. Cases of neurotoxicosis in wildlife are likely under reported due to insufficient surveillance, the initial presumption of rabies or infectious disease, or a lack of funding and access to comprehensive toxicology testing. But such information is necessary for assessing the potential threat consequent of the past use of chlordane in the urban environment.

## 2. Materials and Methods

### 2.1. Clinical Sample Collection and Initial Diagnostic Workup

Five female and twelve male skunks found dead or moribund were submitted to the Michigan Department of Natural Resources Wildlife Disease Laboratory (MDNR WDL) for necropsy (Lansing, MI, USA). The skunks were assessed for age and reported as five adults and twelve juveniles. Brain stems and cerebellums were submitted to the United States Department of Agriculture, Wildlife Services, or the Michigan Department of Health and Human Services (MDHHS) for routine rabies screening. The animals were screened for canine distemper virus and three animals were screened for highly pathogenic avian influenza virus through the diagnostic service at the Michigan State University Veterinary Diagnostic Laboratory (MSU VDL). Liver samples were analyzed for drugs, pesticides, and environmental pollutants using gas chromatography mass spectrometry (GC/MS), based on methods previously described by this laboratory [15,27]. Brain samples were also tested for desmethylbromethalin, a neurotoxic metabolite of the rodenticide bromethalin, by liquid chromatography tandem mass spectrometry (LC-MS/MS). Both brain and liver tissues were quantitatively assessed for organochlorine and PCB concentrations, with confirmed positive findings for chlordane and heptachlor metabolites from mass spectral screening. Control tissues from bovine brain and fat and canine liver, obtained from other necropsy submissions to the veterinary diagnostic laboratory, tested negative for these compounds in GC/MS.

### 2.2. Pesticide Standards

An organochlorine pesticide mix containing alpha-benzene hexachloride or hexachlorocyclohexane (BHC), beta-BHC, gamma-BHC (Lindane), delta-BHC, alpha (cis)-chlordane, gamma (trans)-chlordane, 4,4′ dichlorodiphenyltrichloroethane (DDT), 4,4′ dichlorodiphenyldichloroethylene (DDE), 4,4′ dichlorodiphenyldichloroethane (DDD), aldrin, dieldrin, endrin, endrin aldehyde, endrin ketone, endosulfan I (alpha), endosulfan II (beta), endosulfan sulfate, heptachlor, and heptachlor exo-epoxide was acquired from MilleporeSigma (Sigma-Aldrich Corporation-St. Louis, MO, USA). Oxychlordane, heptachlor exo-epoxide, and heptachlor endo-epoxide standards with chromatographic purity were purchased from Agilent Technologies (Santa Clara, CA, USA). PCB standards for congeners #52, 101, 118, 153, 138, 187, 183, 126, 180, 170, and 209 were obtained from ChemService (West Chester, PA, USA).

### 2.3. Solvents and Reagents

Acetonitrile UV (of a chromatography grade) and isooctane (of a chromatography grade) were purchased from Burdick and Jackson (Muskegon, MI, USA). Hexane, isopropanol, and anhydrous sodium sulfate were purchased from VWR (Radnor, PA, USA). Glacial acetic acid was purchased from Fisher Scientific (Pittsburgh, PA, USA). Pure 100% proof ethyl alcohol was purchased from Koptec (King of Prussia, PA, USA). Solutions of 3:2 (*v*:*v*) hexane–isopropanol, 7.5% sodium sulfate in 18-ohm ultrapure, deionized water, 1% (*v*/*v*) acetic acid in acetonitrile UV, and 80:20 isooctane–ethanol were prepared prior to extraction procedures. The ultrapure water was acquired from a Milli-Q system (MilliporeSigma, Burlington, MA, USA).

### 2.4. Equipment/GC-MS/MS Analysis

A Precellys^®^ Evolution bead mill homogenizer (Bertin Corp, Rockville, MD, USA) was utilized to homogenize all tissue samples. An Agilent Technologies 7890A GC system coupled with an Agilent 7000 GC/MS Triple Quadrupole detector (Agilent, Santa Clara, CA, USA) was used to perform the GC-MS/MS analysis. The GC separation was conducted on a Phenomenex Zebron ZB-Multiresidue column (45 m × 250 μm × 0.25 μm) using a 4 μL injection into an inlet operated in splitless mode (Phenomenex, Torrence, CA, USA). An Agilent 5062-3587 split/splitless glass cylinder with single taper liner was used with the glass wool removed. The inlet pressure was 13.4 psi with an initial temperature of 40 °C held for 1 min, which then increased by 8 °C/minute to 310 °C and was held for 14 min for a total analysis time of 49 min. The helium carrier gas flow rate was 1.2 mL/min.

The triple quadrupole mass analyzer was operated in negative electron ionization mode at 70 eV maintaining a source temperature of 230 °C. Nitrogen served as the collision gas with a flow rate of 1.5 mL/minute and helium was utilized as a quench gas at 2.25 mL/minute. Multiple reaction monitoring was initiated after a 15 min solvent delay. Data acquisition and processing were performed using MassHunter software version 10.2 (Agilent, Santa Clara, CA, USA).

### 2.5. Lipid and QuEChERS Extraction Procedure

Liver and brain tissue were preserved at −20 °C with liver tissues thawed prior to use. Brain tissues were trimmed while frozen to preserve their structural integrity and ensure a representative sample. The following extraction procedure was applied to all samples and also used in the preparation of matrix-matched calibration standards. For lipid fraction assessment, each tissue underwent liquid–liquid extraction. One gram of tissue was combined with 8 mL of 3:2 hexane–isopropanol in a homogenizer tube with 100 μL of a 10 μg/mL PCB 209 internal standard. Matrix-matched standards were prepared with one gram of bovine brain or canine liver previously verified to be free of the analytes of interest. The matrix-matched standards were spiked with 100 μL of a 10 μg/mL PCB 209 internal standard and an appropriate amount of stock or working solution of organochlorine and polychlorinated biphenyls (see below). The tissue was homogenized, and the homogenate was transferred to a 50 mL round-bottom glass tube. Two 8 mL 3:2 hexane–isopropanol washes of the homogenizer tube were added to the round-bottom glass tube before 12 mL of 6.7% sodium sulfate solution in deionized, ultrapure water was added. The samples were vortexed for one minute and centrifuged at 1000× *g* for five minutes. The hexane layer was transferred into pre-weighed 50 mL glass flat-bottom tubes and the solvent was evaporated under a stream of nitrogen with gentle heat in a 40 °C water bath. After cooling to room temperature, the percent fat was calculated. A bovine adipose sample was used for quality control throughout the fat extraction. The samples and matrix-matched calibration standards were reconstituted in hexane and transferred to 10 mL volumetric flasks to a final volume of 10 mL. For pesticide analysis, a QuEChERS extraction was performed using 5 mL of extract and commercially available extraction and clean-up tubes [27]. A final volume of 5 mL of supernatant from the QuEChERS clean-up tube was transferred to a 16 × 125 glass culture tube with 50 μL of dimethylformamide. Samples and standards were dried to completeness under a stream of nitrogen and reconstituted in 500 μL 80:20 isooctane–ethanol.

### 2.6. Calibration Curve Construction and Analysis

Neat standards were prepared by the dilution of a standard stock solution. The working standard concentrations were 1, 10, 100, 200, 500, and 1000 ng/mL in 80:20 isooctane–ethanol. PCB 209 was utilized as an internal standard for PCB quantitation. The samples were diluted in 80:20 isooctane–ethanol in 1:10, 1:100, and 1:1000 preparations as necessary. Matrix-matched standards were similarly prepared in the presence of bovine brain or canine liver that underwent fat and QuEChERS extraction with the addition of pesticide concentrations consistent with the neat standard curve.

### 2.7. Statistical Analysis

Statistical testing was performed in Graphpad Prism (Version 10.2.2; 2024). The numeric concentrations of the summed cyclodienes were converted to categorical data for exposure based on the following scheme: low = concentrations < 1000 ng/g; intermediate = concentrations between 1000 and 9999 ng/g; and high = concentrations > 10,000 ng/g. The high-exposure group was based on established lethal thresholds in avian brains. We applied 10- and 100-fold uncertainty factors to the utilized lethal brain concentration for the intermediate- and low-exposures groups, respectively. This was accordance with exposure assessment guidelines for veterinary toxicologists utilizing a 10-fold uncertainty factor for intraspecies variability and a second 10-fold uncertainty factor to account for interspecies differences in metabolism and physiology [28]. Data were grouped by geolocation as either Wayne County or Other (Clinton, Ingham, Kent, Macomb).

## 3. Results

**Figure 1:**
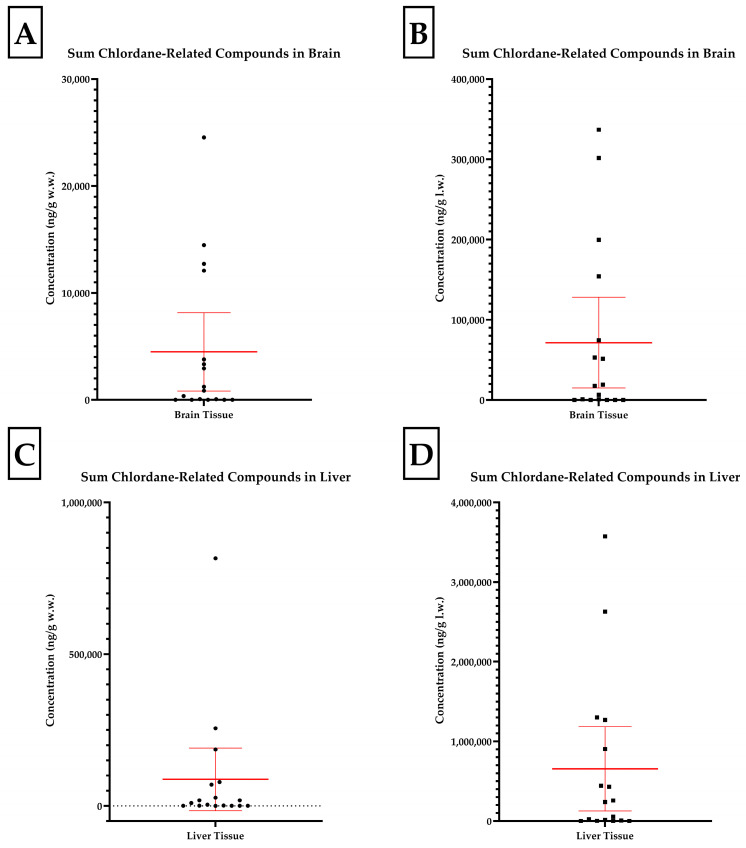
The sum chlordane-related compounds in the brain and liver on a wet weight and lipid weight basis. Concentrations of chlordane-related metabolites in the brain on a wet weight (**A**) and lipid weight (**B**) basis and liver on a wet weight (**C**) and lipid weight (**D**) basis in skunk tissues (n = 17) with a 95% confidence interval of the mean in red.

From July to November 2022, seventeen skunks were assessed by the MDNR WDL. Thirteen of the skunks (76%) presented for evaluation following observed neurologic signs including an inability to balance, altered mentation, tremors, and convulsions or were found dead. One animal (D) had signs consistent with upper respiratory infection. Three additional animals (O, P, and Q) were submitted with nonspecific illness. Complete physical examinations by a licensed veterinary professional were not available and clinical signs were reported by either the Michigan Humane Society intake staff, MDNR Wildlife Division staff, or property owners where the animal was found. There were no significant findings on gross postmortem evaluation. Brains were submitted for histologic examination for nine skunks; three (F, J, and N) were reported to have mild to moderate lymphoplasmacytic encephalitis or meningoencephalitis and the remaining six brains had no significant findings. Initial diagnostic workup ruled out rabies virus, canine distemper virus, highly pathogenic avian influence virus, and bromethalin exposure. Additionally, drugs of abuse, strychnine, carbamate and organophosphorous pesticides, and other toxicants detected by GC/MS were not observed in the samples. Fat content ranged 4.47–10.48% in the brains and 2.97–22.83% in the livers (Table 1). Of the skunk liver samples, twelve (71%) were positive for at least one chlorinated pesticide in GC/MS on initial screening. The concentrations of the three most abundant analytes and summed chlordane related compounds are presented in Table 2. The distributions of the summed chlordane compound data are presented in Figure 1. All analyte concentrations are reported on a wet (w.w.) and lipid weight (l.w.) basis.

**Figure 2:**
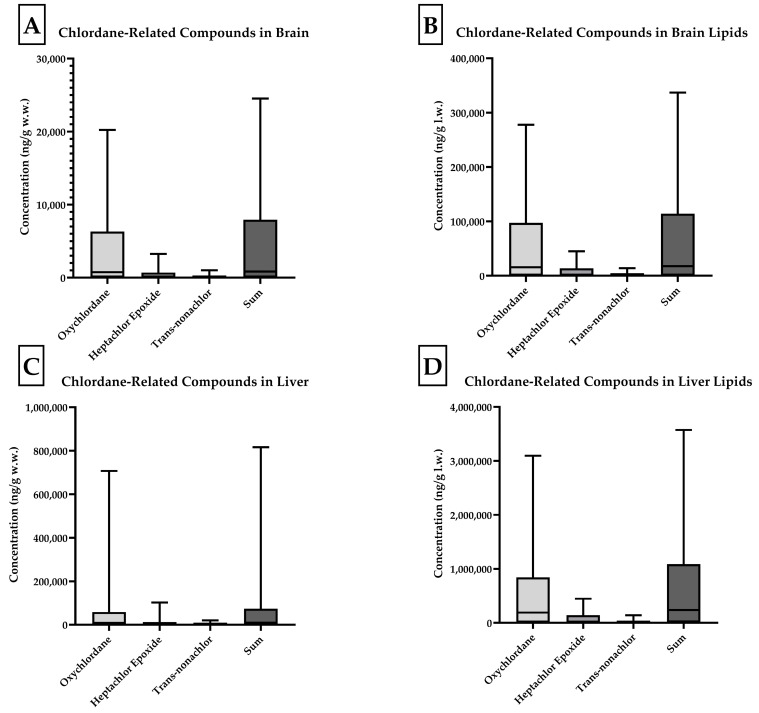
Chlordane-related compounds in the brain and liver on a wet weight and lipid weight basis. Concentrations of oxychlordane, heptachlor epoxide, trans-nonachlor, and total chlordane components in the brain on a wet weight (**A**) and lipid weight (**B**) basis and liver on a wet weight (**C**) and lipid weight (**D**) basis in skunk tissues (n = 17).

Four skunk brains did not contain individual analyte concentrations at or above the 10 ng/g reporting limit (J, O, P, and Q). Of all compounds analyzed, concentrations of oxychlordane, heptachlor epoxide, and trans-nonachlor were present at the highest concentrations. Oxychlordane was present with the greatest abundance in the skunks with the highest total chlordane burden, as noted in Figure 2. Skunks D, H, and M were found to have brain oxychlordane residues that exceeded the 10 µg/g (10,000 ng/g) w.w. lethal residue reported by Stickel et al. for birds [11]. The brain tissue of skunk C contained oxychlordane residues of 9185 ng/g w.w., which was considerably increased with respect to previously reported lethal thresholds for birds; however, the additional brain burden of heptachlor epoxide with a concentration of 2515 ng/g w.w. suggested a potentially lethal burden of chlordane metabolite in this animal’s brain [11]. The remaining eight skunks had total chlordane, nonachlor, and metabolite concentrations ranging from 62 to 3782 ng/g w.w. in the brains. The numeric concentrations of the summed cyclodienes in the brain tissues (w.w.) were converted to categorical data for exposure based on the following scheme: low- (<1000 ng/g), intermediate- (1000–9999 ng/g), and high- (>10,000 ng/g) exposure groups and county of origin (Wayne versus other counties). Chi-square tests for trends analysis revealed a statistically significant trend (*p* = 0.018); however, Fisher’s exact test (*p* = 0.073) did not find an association between concentrations and represented counties. This was likely due to the small sample size of fewer than twenty animals.

**Figure 3:**
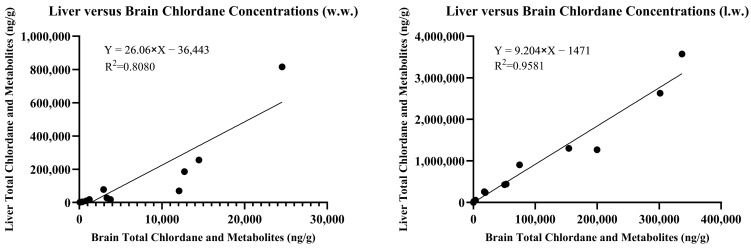
Liver versus brain total chlordane plus metabolite concentrations (ng/g) on a wet weight (w.w) (**left**) and lipid weight (l.w.) (**right**) basis.

The concentrations of chlordane metabolites and trans-nonachlor were significantly greater in the liver tissue when compared to those in the brain tissue from the same individual. The summed chlordane, nonachlor, and metabolite concentrations in the liver tissues ranged from 34 to 815,826 ng/g w.w. and at least one analyte was detected in all but one individual. Seven animals were reported to have total liver residue concentrations less than 1000 (range 34–986), two less than 10,000 (3477 and 9232), five less than 100,000 (range 17,917–78,258), and three greater than 100,000 ng/g w.w. (range 185,648–815,826). When analytes were reported on a lipid weight basis for the brain and liver, the liver total chlordane and metabolite concentrations were approximately ten-fold greater than the brain concentrations. A simple linear regression comparing brain and liver concentrations on a lipid basis yielded an r^2^ = 0.9581 compared to wet weight concentrations with r^2^ = 0.8080, as noted in Figure 3.

**Figure 4:**
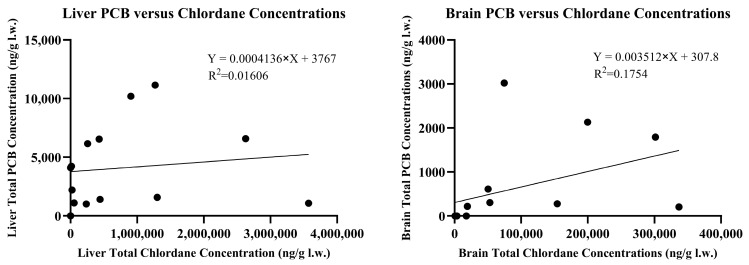
Total PCB versus chlordane and chlordane metabolite concentrations in liver (**left**) and brain (**right**) l.w.

Low concentrations (<500 ng/g w.w.) of dieldrin were detected in 24% of brains and 59% of livers. Two liver samples, H and M, had dieldrin concentrations of 887 and 2417 ng/g w.w., respectively. Additionally, low concentrations (<500 ng/g w.w.) of DDE were detected in 47% of brains and 41% of livers. Skunks H and M also contained DDE concentrations of 619 and 1453 ng/g w.w. in the brain tissue. Five other skunks had hepatic DDE concentrations ranging from 706 to 6806 ng/g w.w. while skunk H contained 11,975 ng/g w.w. DDE. DDD was detected in trace amounts in the liver of skunks C and I and at concentrations of 1019 and 717 ng/g w.w. in skunks H and M, respectively. DDD was not detected in brain tissue of any skunk at concentrations exceeding the 10 ng/g reporting limit. PCBs were detected in 47% of brain samples and 94% of liver samples. In the brains, total PCB concentrations ranged from 14 to 135 ng/g w.w. or 206–3021 ng/g l.w. in the eight positive samples. Liver total PCB concentrations ranged from 19 to 640 ng/g w.w. or 510–11,138 ng/g l.w. in the sixteen positive samples. Negative samples did not have individual PCB congener concentrations exceeding the 10 ng/g reporting limit. PCB congeners #52, 101, and 126 were not detected in any of the seventeen brain or liver samples. Congeners #153, 138, and 180 were found in the highest concentrations in order of decreasing abundance. The accumulation of chlordane and its metabolites was not correlated with the accumulation of PCB congeners in either liver or brain tissue with r^2^ values of 0.0161 and 0.1754, respectively, as described in Figure 4.

## 4. Discussion

**Figure 5:**
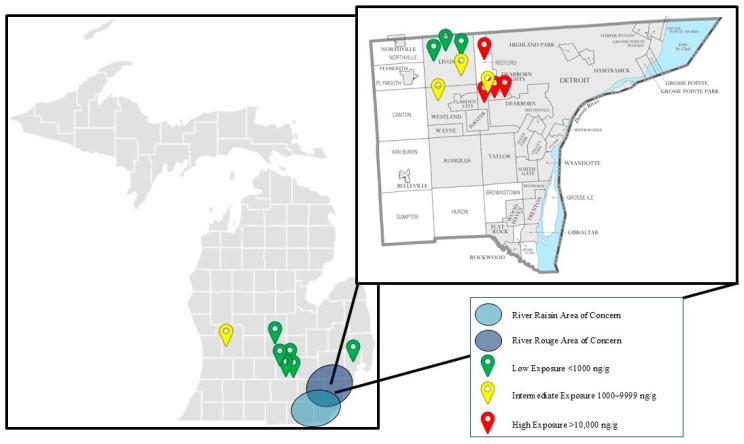
Skunk geographic distribution by summed brain chlordane compound exposure level (wet weight). Summed oxychlordane, heptachlor epoxide, and trans-nonachlor concentrations are divided into three levels of exposure: low (<1000 ng/g), intermediate (1000–9999 ng/g) and high (>10,000 ng/g). Individual animals are represented by pins on the map of Michigan based on their exposure level and county of origin. The River Raisin and River Rouge Areas of Concern are highlighted with shaded ovals overlapping the counties that contain these watersheds.

Ten out of the seventeen skunks (59%) were submitted from Wayne County, Michigan, which encompasses the majority of the River Rouge Watershed. This region is known for its historical contamination with PCBs and other pollutants (Figure 5) [4]. The River Raisin and River Rouge Watersheds span the greater Detroit Metropolitan Area across Wayne, Oakland, and Washtenaw Counties and are the most urbanized and densely populated watersheds in the state of Michigan [1,2,3,4]. Designated as an EPA AOC under the Great Lakes Water Quality Agreement of 1987, the River Rouge prompted the MDNR and Michigan Water Resources Commission to adopt a 20-year remedial action plan beginning in 1985 [2,4]. The river’s sediment contamination with PAHs, PCBs, oil, grease, and heavy metals has led to ongoing monitoring studies, beneficial use impairments, and concerted clean-up efforts [4].

PCBs, dieldrin, and DDE are often analyzed together and are expected to co-accumulate due to a common source of exposure in contaminated watersheds or through bioaccumulation within aquatic food webs [21,29,30,31,32]. Of interest to this study, the accumulations of PCBs, dieldrin, and DDE were not well correlated within brain nor liver tissue. Within the brain, the correlation coefficients of simple linear regression analysis were the following: dieldrin vs. DDE R^2^ < 0.01; PCBs vs. DDE R^2^ = 0.5669; and PCBs vs. dieldrin R^2^ < 0.01. Similarly, the correlation coefficients of simple linear regression in the liver were the following: dieldrin vs. DDE R^2^ = 0.0128; PCBs vs. DDE R^2^ = 0.2280; and PCBs vs. dieldrin R^2^ = 0.0379. While dieldrin, DDE, and PCBs were unlikely to have significantly contributed to the moribund status or death of these skunks, the occurrence of these analytes as persistent organic pollutants in the Detroit Metropolitan Area and their long-term implications for human and ecological health at low concentrations remain a concern.

Lethal thresholds for PCB brain residues were reported as 310,000 ng/g w.w. for birds fed Aroclor 1254 at a rate of 1500 μg/g feed dry weight. None of the skunks reported herein exceeded the lethal threshold for PCB residues in brain tissue with the greatest concentration reported at 135 ng/g w.w. [32]. For comparison, PCB concentrations in Japanese raccoon dogs ranged from 24 to 1200 ng/g l.w. in the liver while PCBs in skunks from this study ranged from 510 to 11,138 ng/g l.w. [17]. The PCB congeners 138, 153, and 180 were found with the greatest abundance and were suggestive of environmental contamination from Aroclors 1260 and/or 1254 [33]. With concentrations 2000 times lower than those reported by Stickel et al., it is unlikely that the PCB residues contributed to the neurologic presentation of these skunks [32]. The finding of PCB residues in skunks residing in areas supplied by the River Rouge Watershed further reinforces that PCBs contaminate and persist in this region nearly 35 years after the remedial action plan was implemented.

Previous reports indicated lethal brain dieldrin concentrations in birds of 10,000 ng/g w.w. and DDE at 500,000 ng/g w.w. in birds [11,34,35], and acutely lethal brain dieldrin concentrations averaged 5500 ng/g w.w. in dogs [36]. Additionally, concentrations of DDE ranged from 5.3 to 150 ng/g l.w. in the liver of Japanese raccoon dogs [17]. Dieldrin and DDE did not exceed previously reported lethal brain concentrations in any of the submitted skunks. The skunks presented herein had hepatic DDE concentrations ranging from 188 to 175,364 ng/g l.w., which suggests either increased dietary exposure or decreased clearance of DDE by skunks when compared to raccoon dogs. These findings of sublethal PCB, dieldrin, and DDE concentrations further support that the neurologic state of these animals was more likely attributed to the combined burdens of oxychlordane, heptachlor epoxide, and trans-nonachlor.

Tissue chlordane and metabolite residue concentrations in the skunks found in urban and suburban areas of Michigan far exceeded previous reports in other small wildlife species. A 2007 analysis of ten raccoon dogs (*Nyctereutes procyonoides*) collected outside Tokyo found hepatic concentrations of oxychlordane ranging from 160 to 20,000 ng/g l.w., concentrations significantly lower than those reported here [17]. The racoon dogs in the study succumbed to traffic accidents; therefore, antemortem clinical signs were not reported [17]. Racoon dogs are opportunistic carnivores consuming small animal prey, fruits, vegetation, and insects, similarly to North American striped skunks (*Mephitis mephitis*), with each species filling comparable ecologic niches [17,23]. Striped skunks are known to inhabit residential areas by building dens around porches, homes, and garages or sheds [23]. Due to the close proximity of skunk dens to the foundations of homes and garages, skunks and other small residential wildlife species have an increased risk of environmental exposure to residues of technical chlordane that was once applied in these locations due to its termiticidal properties [5]. Skunks positive for hepatic oxychlordane residues were found to have concentrations 155 times greater (range 11,318–3,096,515 ng/g l.w.) than those reported in raccoon dogs. While impressive, and suggestive of oxychlordane toxicosis, diagnostic criteria for neurotoxicity and lethality are not available for liver tissue.

There are few available studies specifically investigating the neurotoxic effects of chlordane and its metabolites in mammals. Case studies in humans, for which convulsions and tremors were observed, involved high-dose acute exposures as opposed to chronic environmental or dietary exposure [24,25]. Tremors were reported in rats and mice receiving high-dose chronic oral exposures of chlordane throughout a carcinogenicity bioassay [26]. Previous studies in laboratory animals may not have observed neurotoxic effects due to insufficient dose, short study duration, or the utilization of alternative end points such as feed refusal or severe weight loss [5,37]. This may be due in part to the steep dose–response curve reported by one study in rats which reported an oral toxic dose of 10 mg/kg body weight (BW) and a no-observed-effect-level (NOEL) dose of 1 mg/kg BW with toxicity defined as feed refusal and weight loss [37]. For this reason, postmortem tissue chlordane concentrations are difficult to interpret and diagnostic decision thresholds for neurotoxicity and lethality are not readily available for most species. Female rats receiving 10 mg/kg BW per day of oxychlordane by oral gavage for 28 days were reported to contain adipose tissue oxychlordane concentrations of approximately 600,000 ng/g l.w. and liver concentrations of approximately 18,000 ng/g w.w. [37]. Rats in the 10 mg/kg BW per day dosing group were lethargic and unkempt, but tremors and convulsions were not reported. We report skunk hepatic oxychlordane concentrations up to 707,000 ng/g w.w. (range 517–706,981 ng/g w.w.), nearly 40× greater than those experimentally produced in rats, suggesting a greater dose, repeated exposure, the increased storage efficiency or capacity of oxychlordane, or the decreased elimination of chlordane and related compounds in these skunks.

Three skunks contained brain oxychlordane residues exceeding the 10,000 ng/g w.w. lethal threshold reported for birds [11]. The brain tissue of a fourth skunk contained oxychlordane residues of approximately 9185 ng/g w.w. and was considerably increased in accordance with previous lethal thresholds reported for birds; the additional brain burden of heptachlor epoxide at 2515 ng/g w.w. suggested a lethal brain chlordane metabolite burden for this animal [11]. Therefore, the death or moribund status of these four animals may be attributed to the neurotoxic effects of these analytes. This is further supported statistically as the four animals in the high-exposure group contained summed oxychlordane, hepatachlor epoxide, and trans-nonachlor concentrations exceeding the upper limit of the 95%CI of the mean, suggesting a significant deviation from the population (Figure 1). Moreover, if an uncertainty factor of 10 is utilized to account for species differences, we may consider brain residues of 1000 ng/g w.w. as potentially lethal [37]. Four additional skunks had brain oxychlordane residues exceeding 1000 ng/g w.w. with a fifth considered marginal (753 ng/g). Although the lethal residues described are for oxychlordane individually, the skunks were found to have significant burdens of heptachlor epoxide and trans-nonachlor as well. It is important to reiterate that oxychlordane is six times more toxic when compared to technical chlordane in birds and hepatachlor epoxide is 10 times more toxic than either isomer of chlordane in rats [11,13]. Therefore, the use of comprehensive organochlorine panels and summed chlordane and metabolite burdens provides a more accurate diagnostic approach than analysis of any one single analyte. Data have been presented and discussed throughout on a wet weight basis for ease of interpretation when comparing to previous studies and diagnostic criteria. It is of note that correction for lipid content of tissues and reporting on a lipid weight basis allows for more accurate comparison across tissues within individual animals and within and across species. The liver was shown to be predictive of brain chlordane concentrations on a lipid weight basis and can be utilized to predict neurotoxic chlordane concentrations in the brain when diagnostic samples are limited or brain tissue cannot otherwise be collected during postmortem examination.

While there is strong evidence that toxicologically significant concentrations of chlordane and its metabolites accumulated in the brain tissue of multiple animals, statistically significant differences between groups in this population could not be elucidated. Nevertheless, there is evidence that animals which originated from the Detroit Metropolitan Area had higher brain chlordane concentrations than those from other counties in the state of Michigan (Figure 5). Animals in the highest-exposure group originated from Redford and Dearborn Heights within Wayne County, Michigan. Additionally, one animal submitted from Grand Rapids in Kent County, Michigan, fell within the intermediate-exposure group. A statistically significant difference between population groups when comparing Wayne County to other Michigan counties could not be elucidated. The lack of statistically significant findings in this population were largely due to the small sample size of fewer than twenty animals. The inclusion of additional animals from the western and northern counties as well as the Upper Peninsula of Michigan would provide further insight to the correlation between chlordane metabolite accumulation in densely populated metropolitan areas when compared to rural communities of Michigan. Additionally, due to the circumstances by which animals were submitted for postmortem examination, samples from this population were unlikely to be randomly selected and the population was not normally distributed, violating the assumptions of multiple statistical models. For these reasons, it is not unexpected that a statistically significant difference could not be elucidated despite having strong evidence for toxicologically significant exposures.

This study provides the first report of neurotoxicosis associated with chlordane exposure in skunks across the greater Detroit Metropolitan Area, notably approximately 40 years after the ban of technical chlordane in the United States in 1988. Globally, chlordane compounds were discontinued in 1997 because of their known ability to accumulate in air, soil, and water, to travel long distances from the release location [38], and to have various toxic effects on animals, humans, and the environment. In humans, exposure to chlordane compounds has been linked to type 2 diabetes, lymphoma, obesity, prostate cancer, breast cancer, and lymphoma [39]. Additional non-cancer clinical effects have also been documented and include various neurological signs, including long-term neurological damage, and reproductive effects. Exposure in humans is primarily oral (contaminated food) but also occurs through inhalational and dermal routes [40].

We report alarmingly high concentrations of oxychlordane and heptachlor epoxide, two known chlordane metabolites with neurotoxic potential. This is not surprising considering the Log Kow value of 5.4, the lack of significant abiotic and biotic degradation in soil or water, and effective uptake by organisms in soil and sediments [41]. The identification of persistent contaminants in the Great Lakes Basin and watersheds remains a focus for improved water quality [42]. Even years after use, there is continued concern for water, soil and insect contamination associated with these watersheds that provide habitat and food sources for Michigan’s wildlife and an urban environment for human activities.

## 5. Conclusions

Here, we present the first report of neurotoxicosis associated with chlordane exposure in skunks across the greater Detroit Metropolitan Area in the absence of other common neurologic conditions. A better understanding of the environmental persistence of technical chlordane compounds in soil, water, and insect populations from areas with widespread historical use is critical to the management of this widely found pollutant. We have highlighted the need for more systemic, broad-scale research efforts to monitor technical chlordane in wildlife, domestic animals, and humans in urban settings to understand the nature and extent of pesticide pollution and mitigate risk associated with exposure to these compounds.

## Figures and Tables

**Table 1:** Skunk identifier, demographics, and fat content for brain and liver tissues.

Skunk ID	Sex	Age	City of Origin	County	Brain Fat %	Liver Fat %
A	Male	Adult	E. Lansing	Ingham	8.42	4.17
B	Male	Adult	Westland	Wayne	5.55	17.66
C	Male	Juvenile	Redford	Wayne	6.05	5.49
D	Male	Juvenile	Dearborn Heights	Wayne	4.8	9.73
E	Male	Adult	Okemos	Ingham	10.48	6.39
F	Male	Juvenile	Grand Rapids	Kent	7.5	4.2
G	Female	Juvenile	Livonia	Wayne	6.19	3.38
H	Male	Juvenile	Dearborn Heights	Wayne	8.26	14.27
I	Male	Juvenile	Livonia	Wayne	4.78	3.59
J	Male	Juvenile	Lansing	Ingham	6.55	6.39
K	Female	Juvenile	Dearborn Heights	Wayne	6.39	7.54
L	Female	Adult	Livonia	Wayne	7.3	4.57
M	Male	Juvenile	Dearborn Heights	Wayne	7.29	22.83
N	Male	Juvenile	Livonia	Wayne	4.47	2.97
O	Male	Juvenile	Fraser	Macomb	8.27	5.32
P	Female	Adult	Lansing	Ingham	9.05	14.37
Q	Female	Juvenile	Bath	Clinton	8.17	3.72

**Table 2:** Skunk chlordane and chlordane metabolite concentrations in brain and liver tissue for seventeen skunks.

Analyte	Tissue ^1^	Mean	Std. Dev.	95% CI of Mean	
Oxychlordane	Brain (w.w.)	3770	5890	740	6800
	Brain (l.w.)	85,300	92,400	12,700	108,000
	Liver (w.w.)	73,400	174,000	<10	163,000
	Liver (l.w.)	2,590,000	883,000	93,200	1,000,000
Heptachlor Epoxide	Brain (w.w.)	579	1010	61	1100
	Brain (l.w.)	16,600	14,800	1190	16,400
	Liver (w.w.)	11,200	25,400	<10	24,300
	Liver (l.w.)	113,000	141,000	13,900	158,000
Trans-nonachlor	Brain (w.w.)	155	292	5	305
	Brain (l.w.)	5600	4330	147	4760
	Liver (w.w.)	2590	5300	<10	5310
	Liver (l.w.)	25,000	41,400	818	43,400
Sum Chlordane and Chlordane Metabolites	Brain (w.w.)	4500	7120	836	8160
	Brain (l.w.)	101,000	110,000	15,100	128,000
	Liver (w.w.)	87,200	201,000	<10	191,000
	Liver (l.w.)	697,000	1,030,000	126,000	1,190,000

^1^ All concentrations are reported in ng/g on a wet weight (w.w.) and lipid weight (l.w.) basis. Lipid weight concentrations were calculated by dividing the analyte concentration by the lipid content for each respective animal and tissue type presented in Table 1.

## Data Availability

Data from this study are available upon reasonable request.

## Abbreviations

The following abbreviations are used in this manuscript:

PCB	polychlorinated biphenyl
PAH	polycyclic aromatic hydrocarbon
EPA	Environmental Protection Agency
AOC	Area of Concern
DDT	dichlorodiphenyltrichloroethane
MDNR WDL	Michigan Department of Natural Resources Wildlife Disease Laboratory
MDHHS	Michigan Department of Health and Human Services
MSU VDL	Michigan State University Veterinary Diagnostic Laboratory
GC/MS	gas chromatography mass spectrometry
GC-MS/MS	gas chromatography tandem mass spectrometry
LC-MS/MS	liquid chromatography tandem mass spectrometry
BHC	alpha-benzene hexachloride or hexachlorocyclohexane
DDE	dichlorodiphenyldichloroethylene
DDD	dichlorodiphenyldichloroethane
w.w.	wet weight
l.w.	lipid weight
BW	body weight
NOEL	no-observed-effect level
